# Charcot–Marie–Tooth disease, psychiatric indicators and quality of life: a systematic review

**DOI:** 10.1042/AN20130048

**Published:** 2014-05-27

**Authors:** Joana L.C. Cordeiro, Wilson Marques, Jaime E.C. Hallak, Flávia L. Osório

**Affiliations:** *Department of Neurosciences and Behavior, Medical School of Ribeirão Preto, University of São Paulo, Ribeirão Preto, São Paulo, Brazil; †Technology Institute (INCT, CNPq) for Translational Medicine, Avenida dos Bandeirantes 3900, CEP 14048-900, Ribeirão Preto, São Paulo, Brazil

**Keywords:** Charcot–Marie–Tooth, comorbidity, hereditary motor sensory neuropathy, mental disorder, psychiatry, quality of life, CMT, Charcot–Marie–Tooth, CMTX, X-linked CMT, FSHD, facioscapulohumeral dystrophy, HMSN, hereditary motor and sensory neuropathy, MD, myotonic dystrophy, MS, mental score, OSAS, obstructive sleep apnoea syndrome, PS, physical score, RLS, restless legs syndrome

## Abstract

This study is aimed to conduct a systematic literature review regarding the associations between psychiatric symptoms, functional impairments, and quality of life in patients with CMT (Charcot–Marie–Tooth). The PUBMED, PsycInfo, SCIELO, and LILACS electronic databases were used, and the following search terms were employed: CMT, HMSN (hereditary motor and sensory neuropathy), mental disorder, quality of life, psychiatry, psychiatric, and psychological without the use of time-limit filters. According to the adopted inclusion criteria, 20 studies were included and appraised. These studies indicated that patients with CMT exhibited an increased trend toward depressive symptoms compared with the general population. In addition, CMT patients were exposed to a higher risk of reduced quality of life and significant sleep impairment. Considering the comorbidity of CMT with other psychiatric disorders, the heterogeneity of the instruments used to evaluate the psychiatric symptoms compromised the ability to compare the studies examined. Our results indicate a need for a systematic evaluation of these conditions to minimize the impairments and decreased quality of life caused by CMT.

## Abstract 2

Patients with CMT exhibited an increased trend toward depressive symptoms compared with the general population. In addition, CMT patients were exposed to a higher risk of reduced quality of life and significant sleep impairment.

## CMT (CHARCOT–MARIE–TOOTH): CLINICAL ASPECTS

CMT disease or HMSN (hereditary motor and sensory neuropathy) is the most common inherited disease of the PNS (peripheral nervous system) affecting approximately 1 in 2500 individuals (Skre, 1974). CMT is classified according to its clinical, neurophysiological, inheritance, and genetic patterns (Patzko and Shy, 2012). CMT1 has autosomal dominant inheritance and slow nerve conduction velocity, suggesting a myelin dysfunction; CMT2 has autosomal dominant inheritance and normal nerve conduction velocity, suggesting an axonal dysfunction; CMT4 is recessive and demyelinating; and CMT2-AR is recessive and axonal. Most patients with CMTX (X-linked CMT) have an intermediate nerve conduction velocity.

At least 45 causing genes have already been identified and several others are still coming (Murphy et al., 2012; Patzko and Shy, 2012). The most frequent mutation worldwide is duplication of the PMP22 gene, localized on the 17p11.2–p12 chromosome (Thomas et al., 1997; Marques et al., 2005; Saporta et al., 2011; Murphy et al., 2012), which is responsible about 60–70% of the CMT1 and 50% of the total CMT patients. The second most frequently mutated gene is the GJB1, which is implicated in the CMTX1 group of patients with an intermediate nerve conduction velocity and who occasionally develop abnormalities in the CNS (central nervous system) (Murphy et al., 2012; Patzko and Shy, 2012). MFN2 is the most frequently mutated gene in CMT2 (Murphy et al., 2012; Patzko and Shy, 2012). Interestingly, some of these patients exhibit intellectual deficits (Genari et al., 2011).

The typical CMT phenotype includes onset of predominantly motor length-dependent sensory and motor polyneuropathies within the first two decades of life associated with variable sensory manifestations, decreased or absent tendon jerks, and skeletal abnormalities, such as *pes cavus*, hammer toes, and scoliosis (Thomas et al., 1997; Marques et al., 2005; Patzko and Shy, 2012) However, marked clinical heterogeneity exists, even for the same mutation, in the same family and for identical twins (Marques et al., 1999). Genetic and non-genetic factors must therefore be involved.

Some patients develop a severe disease, resulting in wheelchair or bed restriction and respiratory insufficiency, but most patients develop a slowly progressive disease compatible with a productive life, although their quality of life is almost always compromised.

## CMT: QUALITY OF LIFE AND PSYCHIATRIC COMORBIDITIES

Previous studies have documented that chronic disease has a negative impact on quality of life of CMT patients, both in physical and mental domains (Teunissen et al., 2003; Arnold et al., 2005; Vinci et al., 2005; Padua et al., 2006, 2008a, 2008b, 2008c; Redmond et al., 2008; Boenterd et al., 2010; Calvert et al., 2012).

Similarly, the presence of psychiatric comorbidities is highlighted in these patients and appear negatively associated with the welfare and quality of life, supporting the considerable functional impairments (Rubinsztein et al., 1998; Gemignani et al., 1999; Dematteis et al., 2001; Pfeiffer et al., 2001; Teunissen et al., 2003; Arnold et al., 2005; Vinci et al., 2005; Padua et al., 2006, 2008a; Kalkman et al., 2007; Dziewas et al., 2008; Hattan et al., 2009; Phillips et al., 2009; Boenterd et al., 2010).

Considering the importance of recognizing psychiatric symptoms in patients with neurological diseases, especially with regards to the common etiological factors associated with these conditions, the present study is aimed to perform a systematic review of the literature to assess associations among clinically relevant functional impairments, quality of life, and psychiatric symptoms in patients with CMT.

## METHODS

This review was conducted in accordance with the Preferred Reporting Items for Systematic Reviews and Meta-Analyses–PRISMA (Moher et al., 2009) as well as following the instructions of Cochrane Handbook for Systematic Reviews of Interventions (Higgins and Green, 2011).

**Figure 1:**
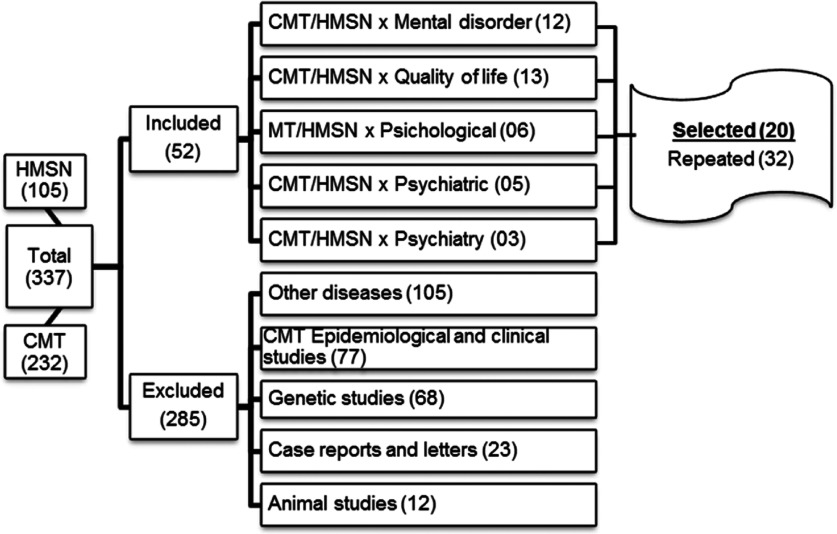
Inclusion/exclusion flow chart for the evaluated studies (HMSH, hereditary motor and sensory neuropathy; CMT, Charcot–Marie–Tooth disease)

The terms CMT, HMSN, psychiatric, psychological, psychiatry, mental disorder, and quality of life were systematically searched using the PsycInfo, LILACS, and PUBMED databases. The inclusion criteria were as follows: (a) language: English or Portuguese; (b) studies examining the psychiatric indicators and quality of life in patients with CMT; and (c) studies without date limit filters. The last search was conducted in January, 2014. The exclusion criteria were as follows: (a) studies related to other neurological disorders; (b) epidemiological studies related to CMT; (c) studies performed in animals; and (d) genetic, clinical, and/or epidemiological studies related to CMT. Figure 1 depicts the selection process used to identify studies.

## RESULTS

Using the search terms and inclusion and exclusion criteria, 20 studies were selected, and their relevance was assessed by a psychiatrist and a clinic psychologist.

### Study designs

Only one study was qualitative descriptive (Arnold et al., 2005). Among the quantitative studies, two referred to themselves as descriptive (Gemignani et al., 1999; Padua et al., 2008a), while the remaining studies employed comparisons among groups (Rubinsztein et al., 1998; Dematteis et al., 2001; Pfeiffer et al., 2001; Teunissen et al., 2003; Vinci et al., 2005; Padua et al., 2006, 2008b, 2008c; Kalkman et al., 2007; Dziewas et al., 2008; Redmond et al., 2008; Hattan et al., 2009; Phillips et al., 2009; Vinci et al., 2009; Boenterd et al., 2010; Calvert et al., 2012; Boentert et al., 2014).

Considering the specific aims of the evaluated studies, it was possible to divide them into two groups, as follows: (a) studies investigating the comorbidity of psychiatric disorders in patients with CMT (Rubinsztein et al., 1998; Gemignani et al., 1999; Dematteis et al., 2001; Pfeiffer et al., 2001; Arnold et al., 2005; Padua et al., 2006, 2008a; Kalkman et al., 2007; Dziewas et al., 2008; Hattan et al., 2009; Phillips et al., 2009; Vinci et al., 2009; Boenterd et al., 2010) and (b) studies evaluating the impact of the CMT symptoms on the quality of life (Teunissen et al., 2003; Arnold et al., 2005; Vinci et al., 2005; Padua et al., 2006, 2008a, 2008b, 2008c; Redmond et al., 2008; Boenterd et al., 2010; Calvert et al., 2012; Boentert et al., 2014), resulting in the current clinical presentation.

The most of the studies were conducted in Europe, especially in Italy (N=7) (Gemignani et al., 1999; Vinci et al., 2005; Padua et al., 2006, 2008a, 2008b, 2008c; Vinci et al., 2009), suggesting a lack of studies in the Americas and Eastern countries. The distinguished cultures can distinctly contribute to the assessment of the quality of life.

### Sample characteristics

Ten studies evaluated the different forms of CMT (CMT1, CMT2, CMTX, and other forms of CMT) (Gemignani et al., 1999; Arnold et al., 2005; Vinci et al., 2005; Padua et al 2006, 2008a; Redmond et al., 2008; Hattan et al., 2009; Vinci et al., 2009; Boenterd et al., 2010; Boentert et al., 2014), nine studies exclusively analyzed patients with CMT1 (Rubinsztein et al., 1998; Dematteis et al., 2001; Pfeiffer et al., 2001; Teunissen et al., 2003; Padua et al., 2006, 2008b, 2008c; Kalkman et al., 2007; Dziewas et al., 2008; Phillips et al., 2009), and a single study analyzed only CMT2 (Teunissen et al., 2003).

The diagnoses were performed using a combination of clinical manifestations, electromyography, and a genetic exam in 85% of the studies.

Regarding nonclinical groups, utilized comparison groups, nine studies employed the general population (Dematteis et al., 2001; Pfeiffer et al., 2001; Teunissen et al., 2003; Dziewas et al., 2008; Hattan et al., 2009; Phillips et al., 2009; Vinci et al., 2009; Boenterd et al., 2010; Calvert et al., 2012) and five studies used normative data (Vinci et al., 2005; Padua et al., 2006, 2008b, 2008c; Redmond et al., 2008). However, seven studies (Rubinsztein et al., 1998; Pfeiffer et al., 2001; Kalkman et al., 2007; Hattan et al., 2009; Phillips et al., 2009; Calvert et al., 2012; Boentert et al., 2014) compared data with clinical studies examining other neurological disorders, such as NH (nonhereditary neuropathies), rLTNCs (rare long-term neurologic conditions), MD (myotonic dystrophy), FSHD (facioscapulohumeral dystrophy), insomnia, and post-stroke patients.

Data are detailed in Table 1.

**TABLE 1:** Sample compositions and countries of origin of CMT disease studies

					CMT forms [*n* (% total)]				
Study No./authors/year	Country	*n*	Control	Age (years)	CMT1	CMT2	CMTX	Other forms	Indeterminate forms
1. Rubinsztein et al. (1998)	England	13	36*	18–70	13 (100)	–	–	–	–
2. Gemignani et al. (1999)	Italy	44	–	60	17 (39)	27 (61)	–	–	–
3. Dematties et al. (2001)	France	11	3^†^	9–72	11 (100)	–	–	–	–
4. Pfeiffer et al. (2001)	Germany	50	23^‡^	17–76	50 (100)	–	–	–	–
			132^§^						
5. Teunissen et al. (2003)	Netherlands	43	1063^†^	17–78	43 (100)	–	–	–	–
6. Arnold et al. (2005)	USA	14	–	32–74	12 (86)	1 (7)	–	1 (7)	–
7. Vinci et al. (2005)	Italy	121	ND	15–78	80 (66)	–	–	41(34)	–
8. Padua et al. (2006)	Italy	195	ND	8–90	153 (79)	14 (7)	16 (8)	12 (6)	–
9. Kalkman et al. (2007)	Netherlands	73	79*	18–60	73 (100)	–	–	–	–
			65^□^						
10. Dziewas et al. (2008)	Germany	12	24^†^	44	12 (100)	–	–	–	–
11. Padua et al. (2008a)	Italy	195	–	8–90	153 (79)	14 (7)	16 (8)	12 (6)	–
12. Padua et al. (2008b)	Italy	137	ND	14–90	137 (100)	–	–	–	–
13. Padua et al. (2008c)	Italy	89	ND	14–71	89 (100)	–	–	–	–
14. Redmond et al. (2008)	Australia	295	ND	18–75	97 (33)	21 (7)	27 (9)	13 (4)	137 (47)
15. Hattan et al. (2009)	Canada	51	173^¶^	61	20 (39)	31 (61)	–	–	–
			245^†^						
16. Phillips et al. (2009)	England	13	35*	18–71	13 (100)	–	–	–	–
			16^†^						
17. Vinci et al. (2009)	taly	53	53^†^	16–64	38 (72)	2 (4)	–	13 (24)	–
18. Boentert et al. (2010)	Germany	227	622^**^	18–78	112 (49)	36 (16)	9 (4)	70 (31)	–
19. Calvert et al. (2012)	Australia	45	26^†^	50	45 (100)^‡‡^	–	–	–	–
			221^††^						
20. Boentert et al. (2014)	Germany	61	61^§§^	22–74	44 (72)	17 (28)			

IF, indeterminate forms; ND, normative data; N, number of subjects with CMT

* Patients with myotonic dystrophy;

^†^ Healthy subjects;

^‡^ Patients followed for 6 months after stroke;

^§^ Healthy elderly, without aphasia or cognitive deficits, according to mini-mental;

^□^Patients with facioscapulohumeral dystrophy;

^¶^NH;

^**^Elderly from German population;

^††^rLTNCs;

^‡‡^Non-informed subgroup;

^§§^Patients with insomnia.

### Assessment tools

The instruments applied to evaluate the clinical variables in the examined studies are schematically presented in Table 2.

**Table 2:** Instruments used for the evaluation of different clinical variables (non-exclusive categories) *According Table 1.

Variable (*n*=number of studies)	Instruments (no. of study) *
Quality of life N=10	SF-36 ^(7,8,11,12,13,14,18)^
	Qualitative interview ^(6)^
	RAND-36 ^(5)^
	EQ-5D ^(19)^
Clinical symptoms *n*=11	
Muscle strength *n*=8	MRC ^(1,8,9,11,12,16)^
	DASH, NASH ^(13)^
	SRT ^(17)^
Deambulation *n*=4	DI ^(8,11)^
	RS ^(4,16)^
	HAIS ^(4)^
Fatigue *n*=4	MFI-20 ^(18)^
	CIS ^(9)^
	FQ ^(1)^
	FSS ^(20)^
Psychiatric disorders *n*=13	
Depressive disorders *n*=8	BDI ^(4,8,9,11)^
	Qualitative interview ^(6)^
	SCID- IV, GHQ-12 ^(9)^
	SDS ^(4)^
	HAD ^(16)^
	SADS-L ^(1)^
	SQ ^(17)^
Anxiety disorders *n*=3	SCID-I-R, GHQ-12, SCL-90 ^(9)^
	HAD ^(16)^
	SQ ^(17)^
Sleep disorders *n*=10	Polysomnography ^(3,10,20)^
	Clinical evaluation made by neurologist ^(2,15)^
	ESS ^(10,16,18,20)^
	PSQI ^(18,20)^
	SCL-90 ^(9)^_,_ SDS,
	SIP ^(4)^
	MHSQ ^(1)^

ESS, Epworth Sleepiness Scale; MFI-20, Multidimensional Fatigue Inventory; BDI, Beck Depression Inventory; DI, Deambulation Index; SIP, Sickness Impact Profile; HAD, Hospital Anxiety and Depression Scale; GHQ-12, 12-Item General Health Questionnaire; CIS, Checklist Individual Strength; MHSQ, Maudsley Hospital Sleep Questionnaire; DASH, Disability Arm Shoulder Hand Questionnaire; NASH, Lumbar Spine Outcome Assessment Instrument; SDS, Self Rating Depression Scale; HAIS, Hauser Ambulation Index Score; RS, Rankin Scale; FQ, Fatigue Questionnaire; SADS-L, Schedule for Affective Disorder and Schizophrenia; MRC, United Kingdom Scale; SQ, Kellner's Symptom Questionnaire; SRT, Symptom Rating Test; SF-36, Study Short Form-36; RAND-36, Health Survey Questionnaire; PSQI, Pittsburgh Sleep Quality Index; SCID-IV, Structured Clinical Interview for DSM-IV; SCL-90, Symptom Checklist-90; FSS, Fatigue Severity Scale; EQ-5D, 5-Question Multi Attribute Questionnaire

Most of the studies used the gold standard instrument for quality of life evaluation, the SF-36 scale (Vinci et al., 2005; Padua et al., 2006, 2008a, 2008b, 2008c; Redmond et al., 2008; Boenterd et al., 2010). This scale is composed of both the PS (physical score) and the MS (mental score). Briefly, while low PSs indicate severe physical dysfunctions, distressing body pain, fatigue, and an unfavorable progression, low MSs indicate frequent emotional suffering and severe social interaction deficits. Higher scores represent a better quality of life. The remaining studies employed qualitative interviews (Arnold et al., 2005) and the RAND-36 (Teunissen et al., 2003) and EQ-5D (Calvert et al., 2012) scales. Concerning the clinical symptoms of CMT, 11 studies (Rubinsztein et al., 1998; Pfeiffer et al., 2001; Teunissen et al., 2003; Padua et al., 2006, 2008a, 2008b, 2008c; Kalkman et al., 2007; Phillips et al., 2009; Vinci et al., 2009; Boenterd et al., 2010) evaluated patients using scales that rated the intensity of the primary disease signals and symptoms, such as muscle strength, deambulation, and fatigue. For the evaluation of muscle strength, there was a trend toward the employment of the United Kingdom Scale (MRC) (Rubinsztein et al., 1998; Padua et al., 2006, 2008a, 2008b; Kalkman et al., 2007; Phillips et al., 2009). In contrast, there was a considerable degree of variation in the instruments used to evaluate deambulation, fatigue, and sleep disorders.

Among the studies that evaluated the occurrence of psychiatric disorders, a single study (Kalkman et al., 2007) used the Structured Clinical Interview for DSM-IV, the gold standard instrument for psychiatric diagnoses (Del-Ben et al., 2001). The instruments used by the other studies were based on self-rating scales that only estimated signals and symptoms, which are not adequate for the diagnosis of mental disorders. This is an important limitation of these studies.

### CMT and quality of life impairment

The primary results related to quality of life in patients with CMT are presented in the Table 3.

**Table 3:** Indicators of quality of life and psychiatric disorders in patients with CMT, non-exclusive categories *According Table 1.

Variable (*n*=number of studies)	Instruments (No. of study)*
Quality of life impairment *n*=10	CMT>Cs, *n*=8^(5,7,8,12,13,14,18,19)^
	CMT<rLTNCs^(19)^
	Positive correlation with muscle strength impairment, *n*=4 ^(8,11,12,13)^
	Positive correlation with deambulation deficit, *n*=3^(6,8,11)^
	Positive correlation with fatigue, *n*=1^(18)^
Psychiatric disorders *n*=13	
Depressive disorders *n*=8	CMT=Cs, *n*=2^(4,17)^
	CMT > Cs, *n*=1^(16)^
	CMT < MD, *n*=2^(1,16)^
	CMT=MD, *n*=1^(9)^
	CMT=FSHD, *n*=1^(9)^
	CMT=stroke, *n*=1^(4)^
	Positive correlation with reduced quality of life and depressive symptoms, *n*=4^(6,8,9,11)^
	Absence of correlation between depressive symptoms and physical impairments, *n*=2^(4,16)^
	Absence of correlation between depressive symptoms and disease progression, *n*=1^(8)^
Anxiety disorders *n*=3	CMT=MD and FSHD, *n*=1^(9)^
	CMT < MD, *n*=1^(16)^
	CMT < Cs, *n*=1^(16)^
	CMT=Cs, *n*=1^(17)^
Sleep disorders *n*=9	CMT > Cs, *n*=6^(2,3,4,15,16,18,20)^
	CMT=Cs, *n*=1^(10)^
	CMT > stroke, FSHD, NIN, *n*=2^(4,9,15)^
	CMT > MD, *n*=1 ^(9)^
	CMT < MD, *n*=1^(1)^
	CMT=MD, *n*=1^(16)^

Cs, healthy controls; MD, myotonic dystrophy; FSHD, facioscapulohumeral dystrophy; NIN, non-inherited neuropathy (acquired); rLTNCs, rare long-term neurologic conditions (Huntington's disease, cerebellar ataxia, motor neurone disease, multiple system atrophy, progressive supranuclear plasy, postpolio syndrome); CMT, Charcot–Marie–Tooth disease.

Most studies (N=8) (Teunissen et al., 2003; Vinci et al., 2005; Padua et al., 2006, 2008b, 2008c; Redmond et al., 2008; Boenterd et al., 2010; Calvert et al., 2012) reported impaired quality of life in patients with CMT compared with healthy control groups, although CMT does not appear to alter the life span.

Most studies have reported that decreased quality of life is associated with muscle strength (Padua et al., 2006, 2008a, 2008b, 2008c), deambulation (Padua et al., 2006, 2008a), and fatigue (Boenterd et al., 2010). That is, the weakened physical condition of CMT patients appears to be the primary factor that contributes to the observed decrease in the quality of life. Additionally, it was expected that the progression of the disease would also decrease the quality of life.

However, the results were controversial. Three studies (Teunissen et al., 2003; Padua et al., 2006, 2008b) did not find any decrement in the quality of life related to aging or the duration of the disease. However, two studies (Vinci et al., 2005; Redmond et al., 2008) that compared data from CMT patients with healthy controls using the SF-36 scale revealed a significant difference associated with age, with quality of life assessments reaching higher rates in elderly patients with CMT. This discrepancy could be due to the subjective evaluation of the quality of life, according to Shy and Rose (2005) which is dynamic and can vary during the disease time course. Additionally, the slow progression of CMT allows patients with CMT to find ways to adapt and reduce the impact of the disease on their quality of life.

Studies that employed the SF-36 scale to evaluate patients suffering from other chronic diseases, such as diabetes mellitus (Tapp et al., 2006), stroke (Mayo et al., 2002), rheumatoid arthritis, COPD (chronic obstructive pulmonary disease), angina, asthma, and epilepsy (Stavem et al., 2000) have shown a decrement in the quality of life compared with healthy subjects. This result is in line with the studies examining CMT patients, suggesting that the observed impairment in the quality of life is not disease-specific but may be related to chronic diseases. These studies did not evaluate the association between the quality of life indicators and the duration of the disease.

### CMT and depressive disorders

There were no consensus results regarding depression in CMT patients compared with control groups (see Table 3). Three studies compared the prevalence of depressive disorders in patients with CMT to that of healthy controls. Two studies (Pfeiffer et al., 2001; Vinci et al., 2009) did not report any significant differences between CMT patients and the control groups. One study (Phillips et al., 2009) found a greater diagnostic of depression in patients with CMT. The other two studies (Pfeiffer et al., 2001; Vinci et al., 2009) did not report any significant differences between CMT patients and the control groups. Importantly, each study used distinct instruments to evaluate of the signals and symptoms of depression.

Despite the small number of studies (Rubinsztein et al., 1998; Pfeiffer et al., 2001; Kalkman et al., 2007; Phillips et al., 2009), patients with CMT appear to display similar indicators of depressive disorders as are observed in other neurological diseases, such as FSHD (Kalkman et al., 2007) and stroke (Pfeiffer et al., 2001). When comparing patients with MD and CMT, the results have been contradictory. While one study (Kalkman et al., 2007) reported the same rates of depression in patients affected by these diseases, two studies (Rubinsztein et al., 1998; Phillips et al., 2009) described a higher prevalence of depression in patients with MD. In all three of these studies, patients with MD or CMT presented higher rates of depressive symptoms than the healthy control groups. The diversity in the sample sizes of patients with MD and CMT, as well as in the methodological issues (self-rating scales–Rubinsztein et al., 1998; Phillips et al., 2009;–versus gold standard instruments–Kalkman et al., 2007), should be emphasized.

The deficiencies or flaws in the instruments of evaluation and the lack of standardization across studies that examine the relationship between depression and CMT can critically influence these data. The employment of instruments that track signals and symptoms instead of standard interviews can mask the true prevalence of depressive disorders. Thus, these limitations did not allow for the formation of a solid conclusion.

Previous studies have described the comorbidity between depression and neurological diseases (van der Werf et al., 2001; Carson et al., 2003; Surtees et al., 2003; Lobentanz et al., 2004; Schrag 2004). Following an evaluation of 300 patients with diseases such as Parkinson’s, migraine, epilepsy, and multiple sclerosis, depression was reported in 40% of the sample (Carson et al., 2003). Moreover, a strong association between depression and worsened indicators of health, including physical, social, and work components, was reported.

Our data regarding the association between depression and CMT are in line with the literature. Nevertheless, it is not yet possible to define the role of the depressive symptoms. If secondary, these symptoms would be related to the disease. If primary, they would be etiological. These studies suggest that patients with CMT are a group at increased risk for depressive symptoms, which can be linked both to the disease and to the specific conditions that result from this disease, and thus, CMT patients constitute targets for further research.

### CMT and anxiety disorders

As observed in Table 3, the results were discrepant when anxiety symptoms were evaluated in patients with CMT and MD or in healthy controls. In patients with CMT, generalized anxiety, phobias, posttraumatic stress disorder, and panic were found. However, only a single study (Kalkman et al., 2007) employed the gold standard interview for DSM-IV, while the others (Phillips et al., 2009; Vinci et al., 2009) used psychiatric symptom self-tracking, which may not correspond to psychiatric diagnoses. Then the divergence in data may result from the differences in the sample sizes and/or methodologies (self-rating scales versus structured interview) among studies. No study addressing anxiety and other neuromuscular diseases was found.

### CMT and sleep disorders

Among sleep disorders, OSAS (obstructive sleep apnoea syndrome) (Dematteis et al., 2001; Dziewas et al., 2008; Boentert et al., 2014), RLS (restless legs syndrome) (Gemignani et al. 1999; Hattan et al., 2009; Boenterd et al., 2010; Boentert et al., 2014), daytime sleepiness (Rubinsztein et al., 1998; Pfeiffer et al., 2001; Phillips et al., 2009; Boenterd et al., 2010), and reduced sleep quality (Rubinsztein et al., 1998; Pfeiffer et al., 2001; Kalkman et al., 2007; Boenterd et al., 2010; Boentert et al., 2014) were analyzed. Most studies (Gemignani et al. 1999; Dematteis et al., 2001; Pfeiffer et al., 2001; Hattan et al., 2009; Phillips et al., 2009; Boenterd et al., 2010) found sleep disturbances in CMT patients compared with healthy controls, irrespective of the type of disturbance or the instrument used for evaluation. In two studies (Dematteis et al., 2001; Dziewas et al., 2008), OSAS frequency was found to be higher in patients with CMT compared with healthy controls. Both studies described a positive correlation between the OSAS severity and neurological impairments in patients with CMT. A higher prevalence of RLS in patients with CMT2 compared with patients with CMT1 was reported in two studies (Gemignani et al. 1999; Hattan et al., 2009), even though the prevalence of RLS in CMT patients was higher in all of the studies (Gemignani et al. 1999; Hattan et al., 2009; Boenterd et al., 2010) compared with control groups. Of note, one study (Boenterd et al., 2010) has reported, using multiple analyses of regression, that CMT is a statically significant predictor of reduced sleep quality, regardless of age or gender.

Then, CMT appears to predispose patients to sleep disturbances. In particular, CMT1 patients are predisposed to OSAS development. From a clinical perspective, it appears critical to investigate OSAS in patients with CMT1. Although the association between RLS and peripheral neuropathy is not fully elucidated, a significant association between CMT and RLS was observed in the present study, suggesting that patients with RLS should be investigated for peripheral neuropathy and vice versa, as RLS may be a treatable manifestation of neuropathy (Rutkove et al., 1995). Thus, patients with CMT can benefit from sleep disorder investigations and treatments, if applicable.

## CONCLUSION

Patients with CMT present a higher risk of developing psychiatric disorders, especially depression. Additionally, these patients appear to be more susceptible to alterations in the quality of life, which can be dramatically influenced by physical limitations. There is a remarkable correlation between sleep disorders and CMT. These data suggest the importance of the systematic evaluation of these conditions to reduce the CMT-induced impairments, as well as the quality of life decrement.

However, our results should be interpreted with caution because the statistical analysis (meta-analysis) could not be performed due to the diversity of instruments employed to assess the distinct outcome variables. Thus, future studies must demonstrate homogeneous methodologies to statistically confirm the clinical evidence. The importance of studies that separate the patients with various CMT subtypes and those that use structured interviews to accurately estimate the association between CMT and psychiatric disorders should also be highlighted.
